# Doxycycline for the treatment of nodding syndrome (DONS); the study protocol of a phase II randomised controlled trial

**DOI:** 10.1186/s12883-019-1256-z

**Published:** 2019-03-06

**Authors:** Richard Idro, Ronald Anguzu, Rodney Ogwang, Pamela Akun, Catherine Abbo, Amos Deogratius Mwaka, Bernard Opar, Phyellister Nakamya, Mark Taylor, Alison Elliott, Angela Vincent, Charles Newton, Kevin Marsh

**Affiliations:** 1 College of Health Sciences, Makerere University, P.O. Box 7072, Kampala, Uganda; 2 Centre for Tropical Neuroscience, P.O. Box 27520, Kampala, Uganda; 3 Centre for Tropical Medicine and Global Health, University of Oxford, Oxford, OX3 7FZ UK; 4 Ministry of Health, P.O Box 7272, Kampala, Uganda; 5 Liverpool School of Tropical Medicine, Pembroke Place, Liverpool, L35QA UK; 6 Medical Research Council/Uganda Virus Research Institute and London School of Hygiene & Tropical Medicine Uganda Research Unit, P.O Box 49, Entebbe, Uganda; 7 Nuffield Department of Clinical Neurosciences, John Radcliffe Hospital, University of Oxford, Oxford, OX3 9TH UK; 8 Department of Psychiatry, St John’s College, University of Oxford, St Giles, Oxford, OX1 3JP UK

**Keywords:** Nodding syndrome, Doxycycline, *Onchocerca volvulus*, Randomized controlled trial

## Abstract

**Background:**

Nodding syndrome is a poorly understood neurological disorder of unknown aetiology, affecting several thousand children in Africa. There has been a consistent epidemiological association with infection by the filarial parasite, *Onchocerca volvulus* and antibodies to leiomodin and DJ-1, cross-reacting with *O.volvulus* proteins, have been reported. We hypothesized that nodding syndrome is a neuro-inflammatory disorder, induced by antibodies to *O.volvulus* or its symbiont, *Wolbachia,* cross-reacting with human neuron proteins and that doxycycline, which kills *Onchocerca* through effects on *Wolbachia,* may be used as treatment.

**Methods:**

This will be a two-arm, double-blind, placebo-controlled, randomised phase II trial of doxycycline 100 mg daily for six weeks in 230 participants. Participants will be patients’ ages≥8 years with nodding syndrome. They will receive standard of care supportive treatment. All will be hospitalised for 1–2 weeks during which time baseline measurements including clinical assessments, EEG, cognitive and laboratory testing will be performed and antiepileptic drug doses rationalised. Participants will then be randomised to either oral doxycycline (Azudox®, Kampala Pharmaceutical Industries) 100 mg daily or placebo. Treatment will be initiated in hospital and continued at home. Participants will be visited at home at 2, 4 and 6 weeks for adherence monitoring. Study outcomes will be assessed at 6, 12, 18 and 24-month visits. Analysis will be by intention to treat. The primary efficacy outcome measure will be the proportion of patients testing positive and the levels or titires of antibodies to host neuron proteins (HNPs) and/or leiomodin at 24 months. Secondary outcome measures will include effect of the intervention on seizure control, inflammatory markers, cognitive function, disease severity and quality of life.

**Discussion:**

This trial postulates that targeting O.*volvulus* through drugs which kill *Wolbachia* can modify the pathogenic processes in nodding syndrome and improve outcomes. Findings from this study are expected to substantially improve the understanding and treatment of nodding syndrome.

**Trial registration:**

Registered with clinicaltrials.gov ID: NCT02850913 on 1st August, 2016.

## Background

Nodding syndrome is a complex neurolo**g**ical disorder of unknown aetiology. The disease burden is unknown but it is estimated that there are about 10,000 affected children in eastern and central Africa. Of these, about 3000 live in Northern Uganda [1, 2].

The first reports of nodding syndrome came from Tanzania in the 1960’s [3]. Subsequent cases were reported from Liberia [4], South Sudan [5–7] and Uganda [8, 9]. The disease affects previously normally developing children characterized by bouts of repetitive head nodding (the pathognomonic feature). Symptoms develop in children between the ages of three and 18 years, and initially, the head nodding occurs in association with feeding, a cold breeze or cold weather [7, 10, 11]. Over the years however, this is complicated by multiple types of seizures, malnutrition, cognitive and physical decline [1, 7, 10, 12].

There have been many studies of environmental, infectious, toxic and genetic factors to determine aetiology but the cause remains unknown to date [13–15]. On the other hand, a consistent epidemiologic association has been documented between nodding syndrome and infection with *Onchocerca volvulus* (a filarial nematode) [6, 10, 12, 13, 15]. This parasite has previously also been associated with the *Nakalanga* syndrome (a tropical syndrome characterised by short stature and malnutrition) [16–18]. *O.volvulus* has in addition been associated with generalised epilepsy [19]. However, *O.volvulus* is endemic in many parts of Africa, Latin America and Asia where it causes corneal keratitis (river blindness) but nodding syndrome has only been reported in parts of Africa. *O. volvulus* has also not been observed in brain tissue. If *O. volvulus* is involved in the aetiology of nodding syndrome, alternative mechanisms other than direct parenchymal injury are likely.

We hypothesise that, nodding syndrome is a neuro-inflammatory disorder, caused by antibodies to antigens from either *O.volvulus* or its symbiont, *Wolbachia*, cross reacting with host neuron proteins (HNPs). Antibodies against HNPs such as the voltage-gated potassium channel complex (VGKC-complex) including the leucine-rich glioma inactivated − 1(LGI1) protein and contactin-associated protein-like-2 (CASPR2), the N-methyl-D-aspartate (NMDA), α-amino-3-hydroxy-5-methyl-4-isoxazolepropionic acid (AMPA), gamma amino-butyric acid (GABA)_A,_ GABA_B_ and glycine receptors are recognised in severe epileptic disorders [20]. Preceding infections may play a role, possibly through molecular mimicry or indirectly by inducing cytokines and allowing pathogenic antibodies gain access to the brain (reviewed in [20, 21]). Two pilot studies have specifically demonstrated evidence of possible cross-reacting antibodies in the pathology of nodding syndrome, including to leiomodin and DJ-1 [2, 22].

*O.volvulus* is transmitted by female blackflies of the genus *Simulium.* Infective larvae are injected into the host during a blood meal. These grow reaching maturity 1–3 years later. The adult worms reside in skin nodules where they can live for 10–15 years producing millions of microfilariae. These migrate under the skin and may be ingested by the next fly to continue the cycle [3]. Ivermectin kills the microfilaria but the adult worm has no specific drug treatment. However, antibiotic depletion of *Wolbachia,* the *O.voluvulus* symbiotic bacteria, with tetracyclines such as doxycycline, results in sterilisation and premature death of the adult worm and marked reduction in dermal microfilaria [23]. The outcome of nodding syndrome could potentially be improved with such a therapy that kills adult *O.volvulus*.

This study aims to examine the effects of treating patients with nodding syndrome, aged 8 years or older, with oral doses of doxycycline 100 mg daily for six weeks compared to placebo, on serum levels of antibodies to HNPs (VGKC complex, leiomodin, DJ-1 and others to be identified in a concurrent case-control study) at 24 months and on seizure control, disease severity, quality of life, ongoing inflammation and microfilaria density. It will also assess safety, adherence to treatment and factors associated with non-adherence.

## Methods

### Study design

This will be a two-arm double-blind parallel group, placebo-controlled phase II randomised trial of oral doxycycline 100 mg daily for six weeks, versus placebo, for the treatment of nodding syndrome.

### Setting

The study will be conducted in the nodding syndrome affected districts of northern Uganda and will be based in Kitgum General Hospital. The setup of this trial has been described elsewhere [24].

### Participants

Participants will be children and adolescents with confirmed nodding syndrome as defined by the WHO [25] i.e. A child who has experienced head nodding on two or more occasions, observed by a trained health worker or documented on EEG, with symptom onset between the ages of three and 18 years, plus any one of 1) triggered by food or cold weather; 2) presence of other seizures or neurological abnormalities and cognitive decline; 3) clustering in space or time in the local population.

### Sample size

From our pilot studies, ~ 50% of patients with prevalent nodding syndrome had antibodies to a HNPs and ~ 60% to leiomodin [2, 22]. We hypothesise that among patients with new onset nodding syndrome (symptoms < 12 months), six weeks’ therapy with doxycycline will cause a 50% reduction in the proportion of patients with and the levels or titres of antibodies to HNPs (a reduction from 50 to 25% compared to placebo) at 24 months. In Uganda, however, there has been no incident cases of nodding syndrome since 2014. It is anticipated that the intervention may not be as efficacious in patients with established symptoms (i.e. patients with symptoms ≥12 months) and the effect on antibodies would be smaller (an anticipated 40% reduction from 50 to 30%). To demonstrate this effect at 80% power and 5% level of significance and allowing for 10% losses to follow up, we will need to recruit a total of 230 patients with established symptoms (115 in either arm).

### Study procedures

**Fig. 1:**
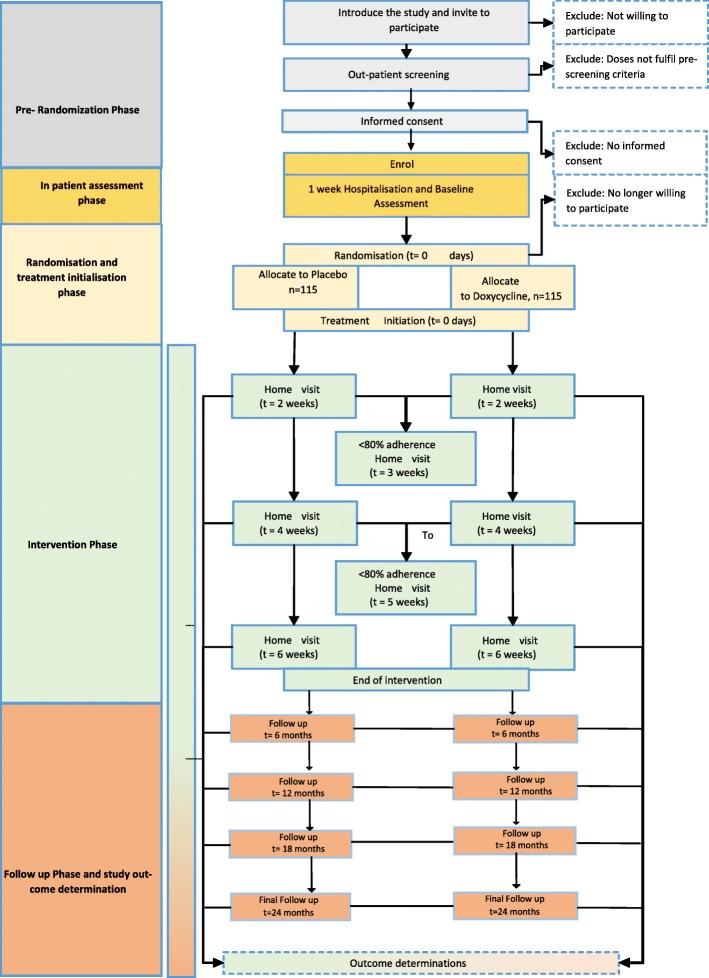
Study design, flow and participant follow up and assessments. Figure is a schematic diagram that shows a time schedule of enrolment, interventions, assessments, and visits for participants

Potential participants will be evaluated through a pre-randomisation phase involving outpatient screening, obtaining consent and enrolment. Consented participants will undergo an inpatient assessment phase, a randomisation and treatment initiation phase, an intervention phase and finally, a follow up and outcome determination phase, Fig. 1.

### Pre-randomisation phase

Potential participants will be identified in specialised nodding syndrome outpatient clinics or community outreach centres. Screening, assent and informed consent procedures will be performed in the same clinics and the community outreach centres by study staff. The inclusion criteria will be patients with confirmed nodding syndrome as defined by the WHO [25] and aged 8 years or older. Females with a positive urinary HCG (pregnancy) test (tested if the participant has experienced menarche), those anticipated to migrate away from the study catchment area, those with known hypersensitivity to tetracyclines, and those with suspected high likelihood of non-compliance to the study drug or follow-up schedule will be excluded. Patients on phenobarbitone, carbamazepine, or phenytoin will first be weaned off and put on sodium valproate, the standard of care antiepileptic drug, before enrolment.

### Inpatient assessment

After eligibility is confirmed and consent obtained, the subject will be hospitalised for 1–2 weeks in Kitgum General Hospital for baseline assessments. The patients’ treatment will be updated per standard of care protocols. The clinical assessment will include a review of the pregnancy, birth and developmental history, the past medical history, a description of the progressive development of the symptoms of nodding syndrome, and the burden and types of seizures. A detailed physical assessment will also be performed including a neurologic, psychiatric and cognitive examination. The Gross Motor Function Classification System (GMFCS) and the Modified Rankin Scale will be used to describe gross motor abilities. The Mini-International Neuropsychiatric Interview for Children and Adolescents (MINI-KID) [26] will be used to document psychiatric disorders and the disorders classified per the Diagnostic Statistical Manual (DSM)-V criteria; Intellectual Disability will be assessed using the Child and Adolescent Intellectual Disability Screening Questionnaire (CAIDS-Q) [27]; nutritional status will be assessed against WHO 2000 anthropometric standards; cognitive function will be assessed using the Cogstate - a computerised cognitive testing program [28], and the quality of life (QOL) will be assessed using the Quality of Life in Childhood Epilepsy (QOLCE) questionnaire [29].

All participants not on standard of care treatments will be initiated on therapy including sodium valproate for seizures, nutritional, physical and psychological therapy as appropriate. Patients receiving current standard of care treatment but with ongoing seizures will have their doses of sodium valproate optimised to 20-35 mg/kg/day.

Five-10mls of blood will be drawn for standard of care testing (full blood count, liver and renal function tests, and HIV). We will also obtain skin snips for *O.volvulus* microfilaria density (on microscopy and Real Time [RT] PCR) and *Wolbachia* load testing. Lumbar puncture will be performed and 3-4mls of cerebrospinal fluid obtained. Aliquots of plasma, serum and cerebrospinal fluid will be stored for baseline evaluation of study specific tests.

All will have a 30-min diagnostic electroencephalographic (EEG) recording. In addition, an overnight EEG will be obtained in a convenient sample of 45 participants selected by stratified random sampling to reflect the five clinical stages of nodding syndrome [30].

### Laboratory testing

This will include the microfilaria density, determination of plasma or serum levels or titres of *O.volvulus* specific IgG, and plasma or serum and CSF levels or titres of inflammatory markers. Markers of inflammation will be tested by ELISA or using Luminex assays. *Antibodies to HNPs such as GAD and the VGKC complex will be tested by radioimmunoassay and antibodies to* LGI-1, CASPR-2, AMPA, GABA_A_ and GABA_B_, NMDA and glycine-receptors *will be tested by cell based assays as indicated by immuno-histology on rodent brain sections* [21]*.*

### Randomisation, allocation and treatment initiation phase

Once all pre-randomisation assessments are complete, participants will be randomised to either doxycycline or placebo with a 1:1 allocation as per a computer-generated randomisation schedule stratified by skin snip test results in to four groups; A, B, C and D. The randomisation code and stratification will be prepared by an independent statistician at a data centre at Global Health Uganda. The study pharmacy assistant will email the independent data centre with the study ID and the microscopy results of the skin snip for *O.volvulus* and the data centre statistician will allocate the participant to the study arms*.*

Eligibility criteria for randomisation are fulfilment of the enrolment criteria and the dose of sodium valproate rationalised to at least 20 mg/kg/day (maximum 35 mg/kg/day) if the patient still experiences one or more tonic clonic seizures a month. Participants reported to be unable to swallow capsules, those already enrolled (or who will enrol) into another trial, and those who withdrew consent since enrolment, will be excluded from randomisation.

The intervention drugs (doxycycline capsules [Azudox®] and placebo) will be obtained from Kampala Pharmaceutical Industries, Uganda. The active drug and the placebo will be labelled with the study code by Kampala Pharmaceutical Industries in collaboration with the independent statistician. Each Azudox capsule contains doxycycline hyclate equivalent to 100 mg of doxycycline base. In the treatment of *Onchocerca volvulus,* doxycycline is given in doses of 100-200 mg/day for 4–6 weeks [31]. We chose the lower dose of 100 mg/day since most participants will be adolescents many of who will also have wasting. With an average weight of 33 kg, we anticipate this dose will translate to ~ 3 mg/kg/day. The two interventions will be identical in the number of capsules (one) per day and timing. The drug administration procedures will also be identical. Both the caretakers and investigators will be blinded. However, in the event of medical emergency un-blinding of an individual participant to the PI or safety monitoring teams may be conducted. Otherwise, all treatment allocations will only be disclosed after the data is locked and a statistical analysis plan is in place.

### Administration of the trial medication

The intervention drugs will be packaged in capped plastic containers each containing 50 capsules (42 + 8 extra capsules). Treatment will be initiated in hospital and continued at home. The first dose will be administered under direct observation at the clinic. Participants and their caretakers will then be carefully instructed about taking subsequent doses before they return home. Each participant will be visited at home at 2, 4 and 6 weeks for adherence and safety monitoring (detailed below). Parents/carers will be reminded weekly through mobile phone contact to ensure the participants take the assigned interventions.

The product will be taken with water between 6.00–9.00 am and at about the same time each day. Should a carer forget to give the dose at the set time, they will be advised to give it as soon as this is realised and to continue the recommended regimen until the dose is completed. Participants will be observed for at least 30 min after taking the medication. Should they vomit within 30 min of receiving the intervention drug, the full dose will be re-administered. Such vomiting will be documented. On a single day, repeat dosing will be attempted once. The eight additional capsules in each pack are to cater for such eventualities.

### Intervention phase, adherence and safety monitoring

To minimise losses to follow up and aid follow up visits in the home, a contact cell phone number will be recorded prior to discharge and the participants be accompanied home by a home visitor who will also draw a sketch map to document the route home and obtain the Global Positioning Satellite (GPS) data of the home.

During each visit, the home visitor will document adverse events, ensure seizures are documented in a seizure diary, conduct pill counts and counsel on adherence. Participants with < 80% adherence at weeks 2 and 4 will be visited again the following week and have additional counselling. Intake of the study drug on the day of the home visit will be directly observed. Participants will continue to attend the 1–2 monthly non-study clinical visits.

Adverse events (AEs) and serious adverse events (SAEs) will be documented following ICH GCP principles and all SAEs reported to the DSMB and ethics committees. If children are found to be ill during these home-visits they will be referred to the study clinic for further evaluation as part of the unscheduled visits.

### Follow period and study outcome determination

Study participants will be re-evaluated at 6, 12, 18 and 24 months. Only clinical assessments will be performed at 6, 12 and 18 months. At 24 months, however, participants will again be hospitalised for one week for inpatient observation and testing, during which all the pre-randomisation procedures and tests will be repeated to determine study outcomes.

### Unscheduled visit (passive follow-up)

Parents will be instructed to bring the participants to the study clinic for any suspected illness. The ‘chief complaint’ and ‘diagnoses’ will be recorded using a standard list based on the International Classification of Disease (ICD-10) for children. Consent will be sought from parents or guardians of participants who die for a formal post mortem or verbal autopsy if the post-mortem is not possible.

### Concomitant drugs

All concomitant medications taken during the study will be recorded with the indication, dose information, and dates of administration. However, concomitant use of any other tetracycline is prohibited during the 24-month study period. If needed, a macrolide antibiotic will be used. Randomised participants who take prohibited medications will cease participation. Such participants will remain in the trial and will be included in the primary, intention-to-treat analysis, but excluded from the per-protocol analysis. Doxycycline interacts with barbiturates (e.g. phenobarbitone), carbamazepine, cimetidine, hydantoins (e.g. phenytoin), and rifampicin. These drugs decrease the effectiveness of doxycycline and so will not be co-administered. Those requiring rifampicin will cease participation. On the other hand, side effects of other drugs such as digoxin, insulin, methotrexate, warfarin, or theophylline may be accentuated. Patients requiring these medications will be excluded. Doxycycline may also decrease the effectiveness of penicillin. Thus, during the six-week study drug administration period, alternative antibiotics (co-trimoxazole and cephalosporins) will be used if indicated.

### Data collection and management

#### Data collection

Data will majorly be quantitative and will be obtained from clinical observations, interviews, patient maintained logs, neurophysiologic and cognitive testing, brain imaging and testing of tissue samples. Data will be collected and recorded at the point of contact at the health facility or the participants’ home. Cognitive function on the CogState is exclusively electronic and no additional entry required. Follow up data obtained during home visits will also be collected electronically but all the other data will be paper based. Data forms will be checked at the end of each day for completeness and accuracy.

#### Data management

Prior to the start of the study, we will develop standard operating procedures for all documentation. Study personnel will undergo training in procedures and documentation. Updates of this training will again be provided in the second and third years. Paper based data will be double entered into the databases with validation limits. Once data entry and cleaning are completed, any hard copies of case record forms will be stored for a minimum 7 years as per local guidelines.

### Study monitoring and data quality control

Internal monitoring of the study will be performed by Makerere University Clinical Trials Unit in collaboration with the Amsterdam Institute for Global Health and external monitoring by the East African Consortium for Clinical Research Monitors. In each case, at least five quality audits are provided for.

### Assessment of efficacy and safety

#### Primary efficacy outcome

The objective of the trial is to determine if targeting *O.volvulus* through drugs which kill *Wolbachia* can modify the pathogenic processes in nodding syndrome. We will therefore investigate the effects of doxycycline compared to placebo on the presence and titres of serum antibodies to HNPs (identified in the concurrent case-control study) or leiomodin and on inflammatory markers, the microfilaria density and *Wolbachia* load. The primary efficacy measure will be the proportion patients testing positive for and levels or titres of antibodies to HNPs or leiomodin at 24 months.

#### Secondary efficacy outcomes

Key secondary efficacy measures at 24 months will include:

1. Mean change in serum levels or titres of antibodies to HNPs or leiomodin and serum levels or titres of inflammatory markers.
2. Mean change in dermal microfilaria density on real time PCR at 24 months.
3. The proportions of patients achieving seizure freedom (≥1 month without head nodding or convulsive seizures), mean change in Cogstate scores, the proportions of patients with inter ictal epileptiform discharges on diagnostic EEG, proportion of participants with Gross Motor Function Classification System (GMFCS) scores 3–5; and the proportion of participants with mental health disorders on the MINI-KID and intellectual disability on the CAIDS-Q and the proportion of participants with improved Quality of Life.
4. Others will be mean changes in weight-for-age, height-for-age (stunting), and height-for-weight (wasting) Z-scores and proportions of participants with wasting and stunting (Z-scores <− 3 SD)
5. Incidence rate of non-nodding syndrome sick clinic visits and all cause sick clinic visits; incidence rate of all-cause hospital admissions and all-cause mortality

### Secondary safety outcomes

Secondary safety outcome measures will be the incidence rate of serious adverse events and adverse events up to 24 months after enrolment.

### Statistical analysis

Analysis will be by intention to treat. The analysis will compare the proportions of patients testing positive for and the levels or titres of antibodies to HNPs or leiomodin at baseline and at 24 months in both arms. Both crude (unadjusted) and adjusted effect estimates will be computed. The chi square test will be used to compare proportions of children with the different antibodies in the two arms; the Students t-test will be used for means of normally distributed data and the Mann-Whitney U test for skewed data. We will also determine the mean change or reduction in the levels or titres of the antibodies and inflammatory markers with the intervention. Time to event (e.g. to seizure resolution) will be measured from the date of randomisation using Kaplan Meyer Survival analysis and by Cox regression analysis. For the cognitive tests, in addition to classification as impaired or not (in comparison to norms determined from unaffected sibling controls in the concurrent case control study), we will also measure mean improvements in scores. The rates determined will be adjusted for age, HIV infection, weight for height z scores and corrected for multiple testing. We will examine safety (serious adverse events and adverse events) and assess compliance and the factors for poor compliance.

### Interim analyses and criteria for termination of the trial

An interim analysis is planned for when a total of 132 participants have completed the 24-month assessment. This number will be sufficient to demonstrate a 50% reduction in the proportion of participants testing positive for antibodies to HNPs at 24 months compared to baseline at β = 0.80 and α = 0.05. This information will be reviewed by an independent Data and Safety Monitoring Committee (composed of an experienced epidemiologist who will chair the committee, a paediatric neurologist and a general paediatrician) which may recommend stopping the trial on safety rules or efficacy reasons. The Peto-Haybittle rule will be used to stop the trial for efficacy reasons: i.e. to consider stopping the trial early because of efficacy, a *p-value* < 0.001 should be achieved at the interim analysis. Accordingly, the early stopping rules will be: (1) proof beyond reasonable doubt of a difference in primary outcome between the study and control groups and (2) evidence that would substantially alter the choice of treatment for patients. Otherwise, the overall significance level of the final analysis will be *p* < 0.05. *However, in order not to miss an early but clinically relevant improvement in secondary outcomes and in particular, seizure control, Makerere University School of Medicine Research and Ethics Committee recommended that the first interim analysis should be conducted when 132 participants complete the 12-month assessment. If significant differences fulfilling the Peto-Haybittle rule are observed at this stage, the trial may be stopped.*

## Discussion

The goal of this study is to understand the pathogenesis of, and to develop specific interventions for, nodding syndrome. Currently, there is no specific treatment for nodding syndrome. The only available treatment is a package of therapies aimed at symptomatic relief [32]. This project has the potential to advance the understanding of nodding syndrome, allow definitive diagnosis and gain insight into potential new approaches for treatment and prevention. If evidence of neuro-inflammation is found and/or specific autoantibodies, immune-modulatory therapies, for example with high dose steroids, may be considered at symptom-onset [2]. In addition, a biologic association between nodding syndrome and *O.volvulus* would allow escalation of treatments and prevention. If a potential use for doxycycline, as treatment for nodding syndrome is observed, it would also be a proof of principle that treatment is possible in older children. Such a strategy could also be explored for younger newly affected children. It is important to point out that the proposed use of doxycycline is based on its effectiveness as treatment for adult *O. volvulus.* Its success will not depend on *Wolbachia* playing a direct role in the pathogenesis of NS (although it may), rather, it is a test of the broader hypothesis that *O.volvulus* plays a role in the pathogenesis of NS.

The approach of using doxycycline is supported by the facts that (1) *O.volvulus* infection has had the most consistent association with nodding syndrome out of many potential risk factors examined to date [33]; (2) doxycycline is a cheap and safe drug and the only curative treatment for adult *O.volvulus* currently available [23]; (3) a direct intervention has considerable advantages over epidemiological association studies in elucidating causality. This is thus a low risk but potentially high gain approach: a positive result would have great significance in understanding both pathogenesis and offering a hope of treatment, whilst a negative result would still be helpful in indicating that whatever the pathogenesis, a simple approach to reducing *O.volvulus* load may not necessarily offer an advantage.

The trial has as one of its major strengths - the dual benefit of potentially determining both the pathogenesis of nodding syndrome and providing a specific treatment intervention. This is supported by the use of both clinical and laboratory biomarkers either as primary or secondary outcome measures. The main weakness is that with the decline in incident cases who would potentially register most improvements with treatment, the study will have to rely on patients with established symptoms in whom, probably lower degrees of improvements will be observed.

## Abbreviations

AE: Adverse event
AMPA: α-amino-3-hydroxy-5-methyl-4-isoxazolepropionic acid
CASPR-2: Contactin-associated protein-like-2
CRF: Case Report form
DSMB: Data and safety monitoring board
GABA: Gamma amino-butyric acid
GCP: Good Clinical Practice
HIV: Human Immuno-deficiency virus
IB: Investigator’s Brochure
ICH: The International Conference on Harmonisation
IEC/IRB: Independent Ethics Committee / Institutional Review Board
IMP: Investigational Medicinal Product
ITT: Intention to treat
NMDA: N-methyl-D-aspartate
NS: Nodding syndrome
NSPs: Neuron surface proteins
PK: Pharmacokinetic
RBC: Red Blood Cells
SAE: Serious Adverse Event
SAE: Severe adverse event
SD: standard deviation
SOP: standard operating procedure
SSA: Sub-Saharan Africa
SUSAR: Serious unexpected adverse event or reaction
VGKC: Voltage Gated Potassium Channel Protein Complex
WHO: World Health Organisation
